# Acute endurance exercise modulates growth differentiation factor 11 in cerebrospinal fluid of healthy young adults

**DOI:** 10.3389/fendo.2023.1137048

**Published:** 2023-03-22

**Authors:** Martin Schön, Karin Marček Malenovská, Michal Nemec, Nikoleta Alchus Laiferová, Igor Straka, Zuzana Košutzká, Peter Matejička, Peter Valkovič, Jozef Ukropec, Barbara Ukropcová

**Affiliations:** ^1^ Institute of Experimental Endocrinology, Biomedical Research Center, Slovak Academy of Sciences, Bratislava, Slovakia; ^2^ Institute of Pathophysiology, Faculty of Medicine, Comenius University, Bratislava, Slovakia; ^3^ 2^nd^ Department of Neurology, Faculty of Medicine, Comenius University, University Hospital Bratislava, Bratislava, Slovakia; ^4^ Institute of Normal and Pathological Physiology, Centre of Experimental Medicine, Slovak Academy of Sciences, Bratislava, Slovakia

**Keywords:** exercise, cerebrospinal fluid, GDF11, growth differentiation factor 11, running

## Abstract

**Objective:**

Strong evidence supports the benefits of exercise for healthy ageing, including reduced risk of neurodegenerative diseases. Recent studies suggested interorgan crosstalk as a key element of systemic adaptive response, however, the role of specific molecules in mediating exercise effects on the human brain are not fully understood. In the present study, we explored the exercise-related regulation of Growth Differentiation Factor 11 (GDF11) in cerebrospinal fluid (CSF) and blood.

**Methods:**

The samples of serum, plasma and CSF were obtained before and 60min after acute exercise (90min run) from twenty healthy young individuals. Additional serum and plasma samples were collected immediately after run. GDF11 protein content (immunoblotting), body composition (bioelectrical impedance), physical fitness (VO_2_max, cycle spiroergometry) and cognitive functions (standardized computerized tests, Cogstate) were evaluated.

**Results:**

Running decreased GDF11 protein content in CSF (-20.6%. p=0.046), while GDF11 in plasma and serum were not regulated. Two GDF11-specific antibodies of different origin were used to corroborate this result. Individuals with higher physical fitness displayed greater exercise-induced decrease of GDF11 in CSF than those with lower physical fitness (p=0.025). VO_2_max correlated positively with GDF11 in serum (r=0.63, p=0.020) as well as with the exercise-induced change in GDF11 levels in CSF (r=0.59, p=0.042). Indirect measure of blood-brain barrier permeability (i.e. CSF/serum albumin ratio) tended to positively correlate with CSF/serum GDF11 ratio (p=0.060). CSF levels of GDF11 correlated positively with cognitive functions, including working memory, both before and after run (p<0.05).

**Conclusion:**

Running-induced down-regulation of the GDF11 protein in the cerebrospinal fluid of healthy young individuals indicates the potential role of GDF11 in the exercise-induced cross-talk between periphery and the brain.

## Introduction

Average human life expectancy has doubled in most developed countries in the last two centuries (1), however, healthy, disease-free lifespan has not increased as much (2). Understanding the physiology of healthy ageing with subsequent identification of modalities, that have a potential to increase disease-free life expectancy, has therefore emerged as an urgent need to reduce global ageing-related health burden. An excellent tool to study systemic ageing and rejuvenating processes in mice is heterochronic parabiosis (3). Using this technique, it was shown that blood of young mice has a capacity to improve regenerative potential of tissues in old mice, *via* biological factors counteracting aging-related processes such as cardiac hypertrophy (4), muscle structural and functional impairment (5) and cognitive decline (6). Subsequent proteomic analysis identified Growth Differentiation Factor 11 (GDF11) as a rejuvenating factor, capable of stimulating proliferative capacity of stem cells in skeletal muscle, heart and brain, respectively.

GDF11 shares 89% protein sequence homology with another member of transforming growth factor-β superfamily, Growth Differentiation Factor-8 (GDF8; myostatin), well-known negative regulator of muscle growth (7). Importantly, both circulating level of GDF11 and GDF8 decline with ageing in multiple mammalian species (8), while higher serum GDF11 in middle-aged mice was associated with longer life-span (9). Nevertheless, the role of GDF11 and GDF8 in ageing remains controversial and the availability of the reliable methods to identify GDF11 with sufficient specificity remains a limiting factor for both research and clinical exploitation.

Exercise is a powerful physiological stimulus with a potential to prevent and even treat various age-related chronic diseases, enhance resilience against stress and reduce the burden of „diseasome of physical inactivity” (10). Circulating bioactive molecules, mediators of interorgan crosstalk involved in the systemic integrative response, were shown to be specifically regulated by both acute and regular exercise (11, 12) as well as by aging (4, 5). Exercise-regulated bioactive molecules – exerkines (13) - are released from tissues into the bloodstream in response to physical activity, thus significantly contributing to systemic integration of the exercise benefits and healthy ageing. The results of animal studies support the role of exerkines in periphery-brain crosstalk (14, 15), but the evidence from clinical studies is so far fairly limited (16, 17). Expression of GDF11 was shown to be increased by exercise in skeletal muscle of mice (18), and its serum levels were higher in life long exercising individuals (19). The aim of this study was to explore the acute exercise-induced regulation of GDF11 in cerebrospinal fluid and blood of healthy young individuals and to explore inter-relationships between GDF11 levels and physical fitness, body composition and cognitive functions.

## Materials and methods

### Study design and population

The study protocol was described in detail elsewhere (16). Briefly, twenty volunteers underwent examination and material collection after the 90 min run (experimental Day 1) and at the baseline resting conditions (experimental Day 2). Time between experimental days was approximately 4 weeks. All volunteers were young, healthy (no history of chronic diseases or regular pharmacotherapy), physically active (≥1 h of intense, endurance exercise, ≥3x/week) nonsmokers. The participants were asked to refrain from an exhaustive physical activity, caffeine and alcohol forty-eight hours prior to both experimental days and examinations were performed after an overnight fast.

Day 1 (run) consisted of a 90 min monitored (Polar RS300X, Finland) run at the intensity of 75-80% maximal heart rate (HRmax), which was preceded by cognitive testing (computerized cognitive tests CogState & Memtrax). Blood was collected before, immediately after and 60 min after the 90 min lasting outdoor run. Cerebrospinal fluid (CSF) was collected 60min after the run by atraumatic lumbar puncture. During the run, each participant was provided with a drinking water (~3 dcl) to minimize effects of hemoconcentration on protein levels.

Day 2 (baseline/rest) included collection of fasting blood, anthropometric and metabolic (indirect calorimetry) measurements and an atraumatic lumbar puncture to collect CSF.

The assessment of cardiorespiratory fitness (VO_2_max by cycle spiroergometry) was performed on a separate day, one week after the Day 1.

The study design and the analysis strategy of obtained CSF & blood is depicted in detail in the flow chart (**Supplementary Figures 1A, B**).

The study protocol was approved by the Ethics Committee of the University Hospital Bratislava and conforms to the ethical guidelines of the Declaration of Helsinki 2013. Volunteers received detailed information on the study protocol and signed written informed consent prior entering the study.

### Physiological testing

Body composition was measured with (quadrupedal bioelectrical impedance; BF511, Omron, Japan), resting energy expenditure (REE) and metabolic substrate preference (RQ; respiratory quotient) by indirect calorimetry (Ergostik, Geratherm Respiratory, Germany), blood pressure and heart rate by (Dimarson DM-14508, Taiwan) and cardiorespiratory fitness by cycle spiroergometry (PowerCube-Ergo, Ganshorn Medizin Electron GmbH, Germany) and cognitive functions by computerized cognitive tests (CogState and Memtrax). Cognitive testing was performed both before and after 90 min run. Body Mass Index (BMI) was calculated (body weight [kg]/height [m]^2^) and waist circumference measured at the mid-point between the lower border of the rib cage and the iliac crest. Habitual physical activity profile was evaluated by Baecke questionnaire (20).

### Cerebrospinal fluid sampling

Cerebrospinal fluid (5–8 ml) was taken by an atraumatic lumbar puncture technique in the lateral decubitus position using (Sprotte^®^Pencil-Point needle, 22 G). To avoid blood contamination, the first 3 to 5 droplets of clear CSF have not been collected. The samples were immediately centrifuged at 400xg for 10 min at 4°C to remove the contaminating cells, aliquoted, placed on dry ice and stored at −80°C within 1h of collection. Paired CSF samples from both experimental days were obtained from 18 individuals, samples from additional 2 volunteers were taken only on Day 1 (after run). Post-puncture headache was reported in three out of thirty-eight individual punctures (7.9%), with a spontaneous resolution within 1–5 days.

### Biochemical analyses

Biochemical analyses were performed in a certified biochemical laboratory, and included analyses of serum and CSF glucose (ADVIA Chemistry glucose-hexokinase kit), albumin (N antiserum to human albumin) and insulin (ADVIA Centaur Insulin, all from Siemens Healthcare Diagnostics, UK). HOMA-IR was calculated according to formula fasting insulin (mU/l) × fasting glucose (mmol/l)/22.5.

### Immunoblotting and ELISA

Cerebrospinal fluid, plasma and/or serum (20; 2; 1 μL) were mixed with SDS and DTT containing loading dye, incubated (95°C, 5 min), separated on the 10%-SDS-PAGE and electroblotted to PVDF membrane (Millipore, USA). After blocking (intercept TBS, Licor, USA), membranes were incubated overnight with primary antibodies: (i) GDF11 N-terminus specific (rabbit polyclonal antibody, synthetic peptide GDF11 aa32-61, 1:1000, Abcam, ab71347, UK), (sample set A, n=14, **Supplementary Figure 1B**; paired serum samples n=14; paired CSF samples n=12, unpaired CSF samples available only after run n=2), and (ii) GDF11/8 myostatin rabbit monoclonal Ab, synthetic peptide GDF11/8 aa350-407 (C-terminus), 1:1000, Abcam, ab124721, UK) (sample set A, n=14, **Supplementary Figure 1B**; paired serum samples n=14; paired CSF samples n=12, unpaired CSF samples available only after run n=2). To corroborate the findings, GDF11 C-terminus specific monoclonal antibody was used (recombinant human GDF11 aa299-407, 1:1000, R&D systems, MAB19581, USA) (sample set B, n=13; overlap between A & B sample sets: n=7, **Supplementary Figure 1B**). GDF11-C-terminus antibody was used in 5x concentrated CSF samples. Results obtained by GDF11 (N-terminus specific) antibody were used for the association analysis (sample set A). Secondary antibody IRDye 800CW (Li-Cor, USA, 1:10000) was used to visualize protein content (LI-COR, USA). GDF11/8 with molecular weight between 45-50 kDa was recorded with LiCor infrared imaging system and analyzed with Image Studio Lite (Ver 5.2, Li-Cor, USA). Since GDF8 (myostatin) and GDF11 share ~90% sequence identity in the mature, C-terminal signaling domains and ~52% identity in their N-terminal prodomains (21), C and N terminus specific antibodies and antibody recognizing both GDF11 and GDF8 were used.

Levels of adiponectin in serum and CSF were measured by ELISA [Biovendor, Czech Republic], as described in (16).

### Statistical analyses

Endurance exercise effect was assessed by either Student’s paired t-test (two-time points) or multiple comparison testing with ANOVA and Tukey post-hoc test (three time-points) using JMP [version 4.0.4 academic; SAS Institute, USA]. *A priori* power analysis utilizing our pre-existing data on exercise-induced changes in albumin CSF/serum ratio (marker of blood brain barrier permeability) was performed, with α error probability set to 0.05 and power (1-β error probability) to 0.95. Total sample size of 11 would be needed to determine relevant exercise-induced difference in this parameter. Pearson linear correlation analysis model was used. Values are presented as mean ± SEM and the statistical significance was set at p<0.05. Changes induced by 90 min run were calculated as a fold change (ratio between post-run and pre-run levels).

## Results

### Characteristics of the study population

The characteristics of the study population is presented in **Table 1**. Intensive 90 min run induced a delayed decrease in glycaemia 60 min post-exercise, as compared to baseline and immediately post exercise (baseline (resting)) 4.9 ± 0.5 mmol/l, 0 min post exercise 5.2 ± 0.8 mmol/l, 60 min post exercise 4.4 ± 0.4 mmol/l, p<0.001), and an immediate increase of insulinemia and HOMA-IR after run, with a delayed decrease 60 min post-exercise, as compared to baseline and immediately post exercise (insulinemia: baseline (resting)) 5.8 ± 2.7 mIU/l, 0 min post exercise 8.4 ± 5.1 mIU/l, 60 min post exercise 3.5 ± 2.0 mIU/l, p=0.001; HOMA-IR: baseline (resting)) 1.32 ± 0.69, 0 min post exercise 2.00 ± 1.22, 60 min post exercise 0.72 ± 0.46, p=0.001). There was no running-induced change in glycemia and insulinemia in CSF when compared to baseline (resting) state (p>0.1 for all).

**Table 1:** Characteristics of the study population.

Clinical parameter	Mean ± STDV
Sex (M/F)	12/8
Age (years)	26.2 ± 5.0
Body weight (kg)	70.7 ± 7.6
BMI (kg/m^2^)	22.9 ± 2.0
Body fat (%)	20.7 ± 7.0
Muscle mass (%)	37.9 ± 5.6
Visceral fat (%)	4.6 ± 2.0
Waist circumference (cm)	76.6 ± 5.1
Respiratory quotient (VCO_2_/VO_2_)	0.83 ± 0.05
Resting energy expenditure (kcal/24 h)	1608 ± 276
VO_2_max (mlO_2_/kg_BW_/min)	46.0 ± 8.0
HRmax (1/min)	175 ± 13
Hand grip dynamometry (kg)	90.8 ± 17.9
Habitual physical activity (score)	10.4 ± 1.2
Running distance (km)	15.2 ± 2.3

Data are expressed as mean ± STDV. BMI, body mass index; VO_2_max, maximal aerobic capacity; HRmax, maximal heart rate.

Serum lactate increased immediately after the run and near normalization was observed one hour post-run as compared to baseline (baseline (resting)) 1.1 ± 0.6 mmol/l, 0 min post exercise 3.3 ± 2.3 mmol/l, 60 min post exercise 1.6 ± 1.0 mmol/l; p=0.001). Similar pattern was observed for changes in serum albumin (baseline, resting) 45.1 ± 3.1 g/l, immediately post-exercise 49.1 ± 2.9 g/l, 60 min post-exercise 45.8 ± 2.7 g/l; p=0.001) and serum thrombocyte count (baseline (resting) 219 ± 43 10^9^/l, 0 min post exercise 267 ± 31 10^9^/l, 60 min post-exercise 209 ± 58 10^9^/l, p=0.036), which might reflect hemoconcentration present immediately after run. Interestingly, 90 min run increased lactate in CSF of all volunteers (baseline (resting)) 1.2 ± 0.1 mmol/l, 60 min post exercise 1.9 ± 0.4 mmol/l, p<0.001). Total serum creatinkinase increased immediately after run and compared to baseline remained elevated for another hour (baseline (resting)) 3.2 ± 2.4 umol/l, 0 min post exercise 4.5 ± 3.1 umol/l, 60 min post exercise 4.3 ± 2.9 umol/l, p=0.005).

### The effects of 90 min run on GDF11 in CSF and blood

**Figure 1:**
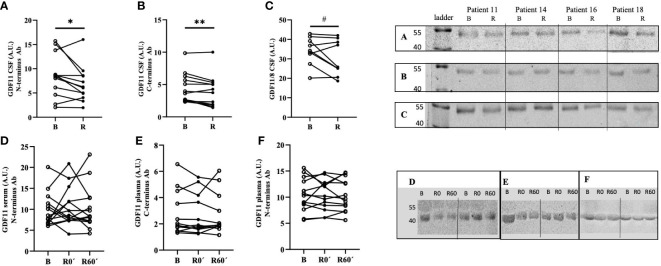
An acute bout of endurance exercise (90 min run) modified the levels of GDF11 in cerebrospinal fluid but not in circulation of healthy trained volunteers. GDF11 levels were assessed by immunoblotting, using three different primary antibodies: **(A)** GDF11-specific N-terminus antibody (CSF, n=12/12, B/R, p=0.046); **(B)** GDF11-specific C-terminus antibody (CSF, n=13/13, B/R, p=0.008); **(C)** GDF11/8 antibody (CSF, n=12/12, B/R, p=0.065); **(D)** GDF11-specific N-terminus antibody (serum, n= 14/14/14, B/0/R); **(E)** GDF11-C-terminus antibody (plasma, n=13/13/13, B/0/R); **(F)** GDF11-specific N-terminus antibody (plasma, n=14/14/13, B/0/R); Due to the lack of space, molecular weight ladders are not visible in **Figures 1D–F** and therefore, the molecular weights of GDF11 are in this case approximate, based on our validation studies of antibodies. B, Basal, before run; R, after run; A.U., signal intensity; GDF, Growth Differentiation Factor. Immunoblotting of all samples was performed under identical conditions. Statistical differences were analysed using paired Student’s t-test. *p<0.05, **p<0.01, #p<0.1 **(A–C)**.

An acute bout of exercise led to the reduction of GDF11 levels in CSF, which was confirmed in paired CSF samples with two GDF11-specific antibodies for C and N terminus, respectively (sample set A, N-terminus antibody: -20.6%, p=0.046, **Figure 1A**; sample set B, C-terminus antibody: -11.2%, p=0.008, **Figure 1B**). Trend towards a decrease in GDF was detected also by antibody against C-terminus which could not distinguish between GDF11 and GDF8 (-10.2%, p=0.065, **Figure 1C**). Thus, the running-induced decrease of GDF11 in CSF was confirmed by 3 distinct GDF11 antibodies. The whole blots are depicted in **Supplementary Figures 2** and **4**. There was no difference in running-induced change of GDF11 levels in CSF of 7 individuals, determined by two different GDF11-specific antibodies (**Supplementary Figure 3**).

Concentration of GDF11 in CSF was lower than that in plasma (-29.7%, p=0.022) or serum (-21.9%, p=0.091). Plasma and serum GDF11 levels did not differ (p=0.261), and not surprisingly there was a positive correlation between GDF11 in plasma and serum (n=55, R=0.376, p=0.003), but not between serum or plasma and CSF levels of GDF11 (data not shown).

Neither serum nor plasma GDF11 levels changed immediately after or 60 min after the run (**Figures 1D–F**). However, there was a decrease in combined protein content for GDF11 and GDF8 in serum one hour after endurance exercise when compared to baseline (-13.4%, p=0.021). Notably, this effect appeared to be gender-specific, as the decrease of serum GDF11/GDF8 in serum one hour after run was significant in women (n=8, -18.5%, p=0.019), but not in men (n=6, -4.9%, p=0.553). This might indicate that the dynamic changes of GDF levels in blood induced by intensive endurance exercise are driven rather by GDF8 than by GDF11. The whole blots depicting dynamics of GDF11 in serum are presented as **Supplementary Figure 4**.

The running-induced changes of GDF11 protein in CSF tended to negatively correlate with changes in its plasma (but not serum) levels (n=11, r=-0.57, p=0.057).

### Effect of an acute bout of exercise on the blood-brain barrier permeability

**Figure 2:**
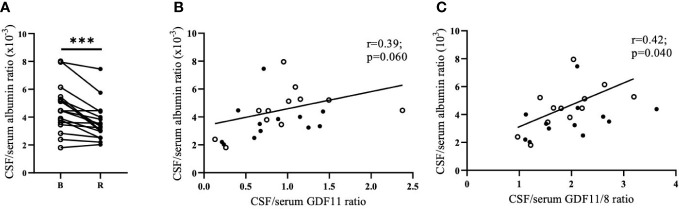
Running-induced decrease of blood-brain barrier permeability (CSF/serum albumin ratio). n=17. (albumin values were not measured in CSF of one individual, and two individuals had CSF available only after the run) **(A)**; its association with CSF/serum GDF11 ratio **(B)**; and CSF/serum GDF11/8 ratio **(C)**. CSF, Cerebrospinal Fluid; GDF, Growth Differentiation Factor. ***p<0.001; Open circles - before run; Full circles - after run.

Blood-brain barrier permeability, as indirectly assessed by the ratio of albumin in CSF and serum, was decreased after the 90 min run by 27.1% (p<0.001; **Figure 2A**). Running-induced changes in CSF levels of GDF11/GDF8 and albumin as well as CSF/serum albumin ratio were positively correlated (n=12, r=0.61/0.66, p=0.036/0.020). Change in GDF11 in CSF tended to positively correlate with a running-induced change in CSF albumin (r=0.54, p=0.069), but not with a change in CSF/serum albumin ratio. CSF/serum albumin ratio tended to positively correlate with GDF11 CSF/serum ratio (n=24, r= 0.39, p=0.060, **Figure 2B**) and positively correlated with GDF11/GDF8 CSF/serum ratio (n=24, r=0.42, p=0.040, **Figure 2C**), indirectly indicating a role of blood-brain barrier permeability in transporting GDF11 and/or GDF8 into the brain.

Albumin CSF/serum ratio correlated negatively with GDF11 levels in serum (n=26, r=-0.40, p=0.042) as well as in plasma (n=26, r=-0.44, p=0.026), but positively with GDF11/GDF8 in CSF (n=26, r=0.50, p=0.009).

### Exercise-induced change in GDF11 levels in CSF is linked to physical fitness

**Figure 3:**
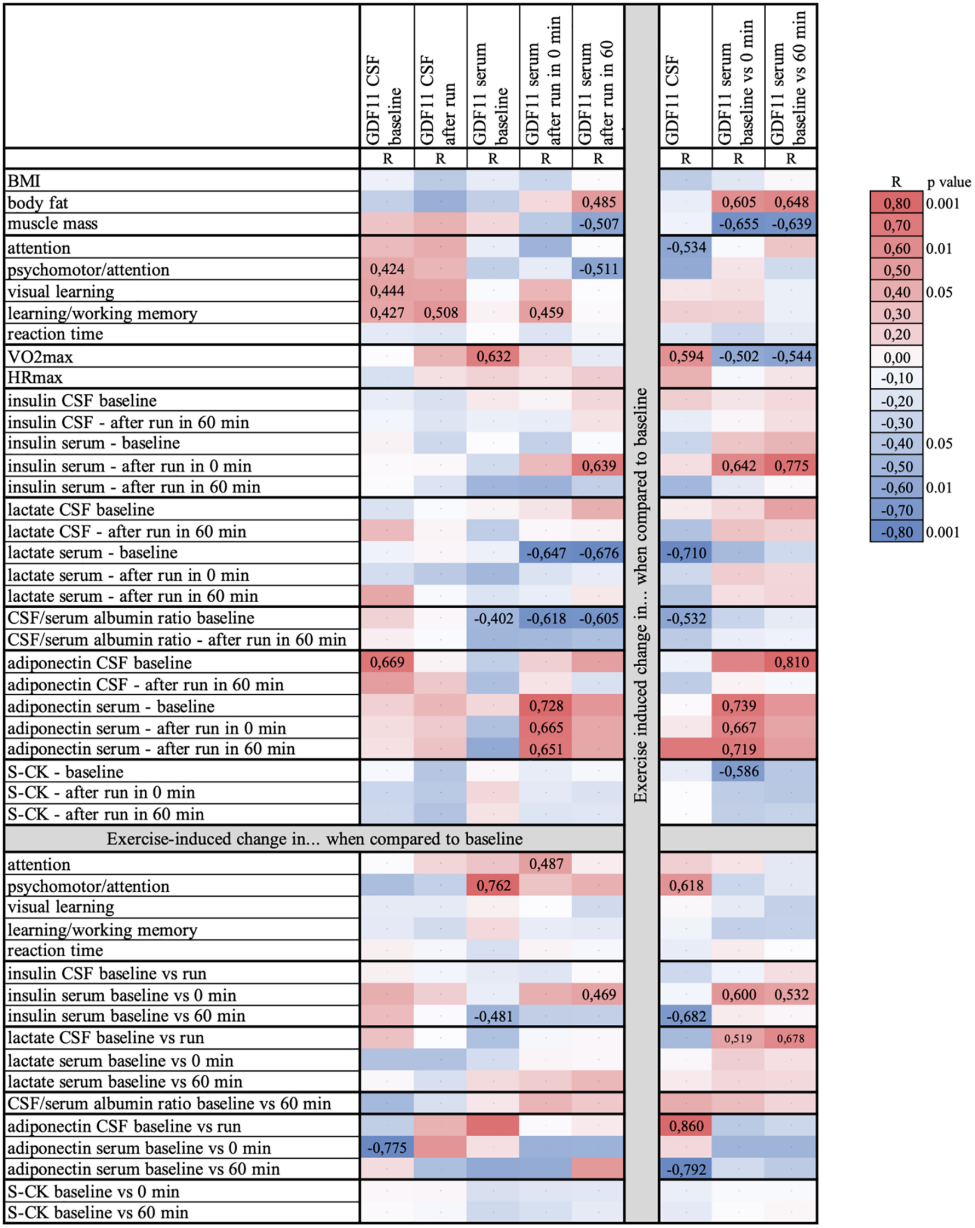
Heat map depicting associations between levels of GDF11 and clinical parameters, as well as their running-induced changes. Data were analyzed using GDF11-specific N-terminus antibody (paired serum samples n=14; paired CSF samples n=12, unpaired CSF samples available only after run n=2). BMI, body mass index; CSF, cerebrospinal fluid; GDF, Growth Differentiation Factor; HRmax, maximal heart rate; S-CK, serum creatinkinase; R, correlation coefficient; VO_2_max, maximal aerobic capacity. Coefficients of significant correlations (p<0.05) or trends (p<0.1) are presented in numbers.

Compared to individuals with higher physical fitness (n=7, M/F 3/4, mean VO_2_max=47.2 mlO_2_/kg_BW_/min, cut off median of VO_2_max=42.2 mlO_2_/kg_BW_/min), individuals with lower physical fitness (n=5, M/F 3/2, mean VO_2_max=35.9 mlO_2_/kg_BW_/min) displayed greater exercise-induced decrease in CSF levels of GDF11 (-34.7% vs. -0.4%; p=0.025). However, there was no fitness-related difference in GDF11 protein content in CSF neither at baseline (resting) state nor after run. The link between physical fitness and exercise-induced regulation of GDF11 in CSF was supported also by a positive correlation between running-induced change in GDF11 levels in CSF and VO_2_max (n=12, r=0.59, p=0.042) (**Figure 3**). CSF levels of GDF11/GDF8 were not associated with physical fitness (data not shown).

Individuals with higher physical fitness decreased, whereas less fit volunteers rather increased GDF11 serum levels measured 60 min after 90 min run compared to baseline (high/low physical fitness; -18.3% vs. 22.5%; p=0.049). This observation was further supported by a trend towards negative correlation between exercise-induced change in serum GDF11 and self-reported habitual sport activity (n=13, r=-0.50, p=0.083). Moreover, baseline GDF11 serum levels positively correlated with physical fitness (n=13, r=0.63, p=0.020) (**Figure 3**). There was no relationship between physical fitness and GDF11 in plasma (p=0.304).

### Associations of clinical phenotypes and GDF levels in CSF and blood

The pattern of associations between GDF11 levels, clinical & biochemical parameters and their running-induced changes is depicted in the heat map (**Figure 3**).

Briefly, there were several associations between GDF11 levels in CSF and cognitive functions assessed by sensitive computerized tests. GDF11 levels in CSF were positively associated with psychomotor performance and attention, visual learning, as well as learning and working memory (p<0.05). Visual memory was also associated with CSF levels of GDF11/GDF8 (r=0.40, p=0.049). On the other hand, running-induced change in serum GDF11/GDF8 levels correlated positively with reaction time in two computerized tests (Memtrax/CogState; r=0.52/0.65, p=0.055/0.012), and negatively with learning and working memory (r=-0.65, p=0.011).

Running-induced change in serum GDF11 was positively associated with body fat (p=0.022) and negatively with muscle mass (p=0.011). Furthermore, it correlated positively with serum insulin immediately after the run (p<0.01) as well as with running-induced change in serum insulin (p=0.024). Running-induced change in GDF11 in CSF correlated negatively with change in serum insulin 60 min after run (p=0.015). Lower serum lactate before run (baseline) was associated with higher GDF11 levels in serum after an acute exercise bout (p<0.05), as well as with a greater running-induced decrease in GDF11 (p=0.01) and GDF11/GDF8 in CSF (p=0.05). Similarly, lower baseline serum creatinkinase was linked with a greater running-induced change in serum GDF11 (p=0.045). Adiponectin, adipokine with a neuroprotective potential which we showed to be regulated by acute intensive exercise (16), was positively associated with levels of GDF11 in CSF, but only at baseline (before run) (p=0.009). Furthermore, there was a positive correlation between running-induced changes in CSF levels of GDF11 and adiponectin (p=0.028). In serum, on the other hand, the positive associations between adiponectin and GDF11 levels or their running-induced changes were present only immediately after the run (p<0.05). Baseline CSF/serum albumin ratio, an indicator of the blood-brain barrier permeability, correlated negatively with GDF11 serum levels before (p=0.042) as well as after the run (p<0.04), with a trend for GDF levels in CSF at baseline (p=0.075).

## Discussion

An acute bout of intensive endurance exercise reduced GDF11 levels in CSF of young healthy physically active individuals. Levels of GDF11 in CSF and serum and/or their running-induced changes were associated with body composition, specific metabolic parameters, aerobic physical fitness and cognitive functions. Previous reports suggested neuroprotective effects of GDF11 in the brain (22) as well as the ability of exercise to modulate GDF11 expression in skeletal muscle and hippocampus (23), proposing GDF11 as one of the mediators of neuroprotective effects of exercise in brain. The vast majority of this evidence, however, comes from animal models, and there is a lack of studies focused on exercise regulation of GDF11 and its potential role in the brain. To the best of our knowledge, this report shows for the first time a direct link between endurance exercise and GDF11 levels in human cerebrospinal fluid.

Exercise-mediated decrease of GDF11 in the brain, with no change in its blood levels, was previously reported in mice (3-month exercise intervention) (23). In humans, short term exercise intervention (6-week high intensity interval training) did not change serum levels of GDF11 in physically active or sedentary humans, but lifelong regular exercise was linked to higher serum GDF11 levels (19).

Initial experiments with heterochronic parabiosis proposed GDF11 as an anti-ageing molecule with an ability to foster rejuvenation (4–6), however, this assumption was later questioned by the study that observed age-related increase in GDF11 levels causing inhibition of muscle regeneration (8, 24). Controversies regarding the role of GDF11 in ageing originate mainly from the absence of a reliable, validated and widely accepted method of GDF11 detection. To support the reliability of our findings as well as to distinguish GDF11 from its close homologue GDF8, we determined GDF11 in CSF, serum and plasma, by immunoblotting, using two different GDF11-specific antibodies, as well as GDF11/GDF8 non-specific antibody. These antibodies have been previously successfully used by the others (18, 25, 26). The reliability of our findings is further supported by correlations between GDF11 in serum and plasma, as well as between GDF11 and serum GDF11/GDF8.

Both caloric restriction and physical activity are known to preserve or enhance cognitive functions and reduce risk of neurodegenerative disorders in humans (27, 28), however the evidence on the molecular mechanisms mediating the neuroprotective benefits of these energy depleting states is relatively sparse, especially in humans. Katsimpardi et al. found that 16-week calorie restriction increased serum GDF11 levels in aged mice, while systemic infusion of GDF11 triggered a calorie restriction-like phenotype and enhanced neurogenic capacity (29). These findings indicate that GDF11 could represent a potential mechanism of neuroprotective rejuvenation, linking anti-ageing effects of calorie restriction and heterochronic parabiosis. In our study, GDF11 levels in CSF were not only regulated by exercise, but they were also associated with several cognitive domains assessed by sensitive computerized tests. It seems therefore plausible that neuroprotective benefits of energy depletion states, such as calorie restriction and exercise, are at least partially mediated by GDF11, a potential mediator of anti-ageing effects of active lifestyle. Reports on animal models suggested neuroprotective actions of GDF11 in the brain. Systemic infusion of GDF11 enhanced neurogenic capacity (29), hippocampal neurogenesis and plasticity (30) in old mice, suggesting GDF11 potential in rejuvenation of aged brain.

We observed physiological changes in serum levels of glucose, insulin and lactate, expected in the population of healthy, young, trained individuals without known metabolic disorder in response to the exercise stimulus (submaximal aerobic exercise). Interestingly, lactate was elevated in CSF of all volunteers one hour after the run (~58%), in contrast to serum lactate which returned to baseline values after an original rapid increase. Thus, dynamic changes of lactate as a marker of exercise exhaustion in serum did not correspond to its changes in CSF. Similarly, there was a distinct exercise-induced dynamics of GDF11 in CSF compared to no change of GDF11 in serum/plasma, the pattern comparable to that of adiponectin, which we have reported previously (16). These observations point at divergent dynamics of exercise-regulated bioactive molecules in the periphery and the brain. It is plausible to speculate that a similar pattern of regulation of GDF11 and adiponectin (16), their mutual relationship as well as their associations with cognitive functions (16) might indirectly indicate their parallel effects on the brain.

Exercise has been shown as an effective strategy to counteract age-related cognitive decline, but molecular mechanisms underlying neuroprotective effects on human brain remain poorly understood (31). To elucidate potential molecular mediators of beneficial effects of exercise in the brain we performed the study in healthy young disease-free volunteers, who participated in 2 experimental days (one including a 90 min run, one resting day). On both days, cerebrospinal fluid and blood samples were taken. Applying this study design, our group have shown that intense endurance exercise (90 min of running) significantly altered levels of adiponectin and other cytokines in cerebrospinal fluid (16) and produced extensive changes in CSF metabolomics specifically related to mitochondrial function and ATP production at the level of the brain (32). In the present study, we report an association between levels of GDF11 and adiponectin in CSF as well as in serum after acute endurance exercise. It was recently shown by Katsimpardi et al. that GDF11 stimulates secretion of adiponectin from white adipose tissue in aged mice, thus repairing neurogenesis in the aged brain (29). These observations support potentially synergic effects of GDF11 and adiponectin on the brain, and the experimental design we implement seems to represent a reliable model to study the regulation of bioactive molecules, potential mediators of neuroprotective effects of exercise in the human brain, by an acute endurance exercise.

The levels of physical fitness was suggested as an important determinant of tissue-specific GDF11 expression, with higher expression found in skeletal muscle, but not in hippocampus, in sedentary old compared to young mice (23). In our report, physical fitness correlated with GDF11 levels in serum, which is in line with results of a previous study reporting higher serum GDF11 in lifelong exercising men compared to their lifelong sedentary peers (19). This study provided the first albeit indirect (correlative) evidence on the putative role of GDF11 in promoting healthy aging in humans, by demonstrating a tight relationship between serum GDF11 and peak power output (19). We extend this observation by showing that the level of physical fitness is an important determinant of regulation of GDF11 by acute exercise, as individuals with lower physical fitness displayed greater exercise-induced decrease in GDF11 in cerebrospinal fluid, and by demonstrating physical fitness-dependent regulation of GDF11 in serum. It seems therefore plausible to speculate that physical fitness not only determines the tissue-specific expression and concentration of GDF11, but also the magnitude of its exercise-stimulated regulation.

In this work, we confirm in a bigger cohort our previous finding (16) that blood-brain barrier permeability, as assessed by CSF/serum album ratio, is decreased after an acute bout of endurance exercise. We observed a modest positive correlation between CSF/serum albumin ratio and CSF/serum GDF11/GDF8 ratio, with a trend also for GDF11. However, exercise-induced changes of CSF/serum albumin ratio and that of GDF11 or GDF11/GDF8 did not correlate, indicating that there are other factors that could modulate levels of this growth factor rather than the blood-brain barrier permeability. This assumption is supported by a series of experiments performed by Ozek et al. (30) demonstrating that circulating GDF11 does not cross the blood-brain barrier, and proposing that at least some of GDF11 neuroprotective effects result from its acting on the brain endothelial cells.

The limitations of this report mostly originate from the nature of the study design and include smaller sample size as well as a relative heterogeneity of the study population. Taking into consideration anti-ageing properties of GDF11, it would be intriguing to apply this study design to individuals within different age groups as well as in those already diagnosed with an early phase neurodegenerative disease. The nature of the experimental design and potential health risks imposed by lumbar puncture hampered the possibility to explore dynamic changes of GDF11 levels in CSF (e.g. immediately after or 24 hours after the 90-min run). Further studies exploring the regulation of GDF11 by different exercise modalities and using animal models are warranted. Finally, mechanistic explanation of reported running-induced changes in CSF levels of GDF11 remains only speculative and further research is needed to fully elucidate the putative role of exercise in neuroprotective effects of GDF11 in human brain.

## Data availability statement

The raw data supporting the conclusions of this article will be made available by the authors, without undue reservation.

## Ethics statement

The studies involving human participants were reviewed and approved by Ethics Committee of the University Hospital Bratislava. The patients/participants provided their written informed consent to participate in this study.

## Author contributions

MS performed the clinical study, analyzed and interpreted the data, wrote and revised the manuscript. KM performed immunoblotting, analyzed the data, contributed to the manuscript preparation and editing. MN, NA, performed immunoblotting. IS, ZK, PM, PV obtained CSF samples. JU co-designed and performed the study, contributed to data analysis, interpretation, writing and revision of the manuscript. BU developed the idea, designed, coordinated and performed the study, analyzed & interpreted data and wrote & revised the manuscript. All authors contributed to the article and approved the submitted version.
