# Supplementary material for: Acute endurance exercise modulates growth differentiation factor 11 in cerebrospinal fluid of healthy young adults
Source: Front Endocrinol (Lausanne). 2023 Mar 22;14:1137048. doi: 10.3389/fendo.2023.1137048 (PMC10073538; doi:10.3389/fendo.2023.1137048)
Supplement: Supplementary file 1 [file DataSheet_1.docx]

**Supplementary material**

**Supplementary Figure 1**


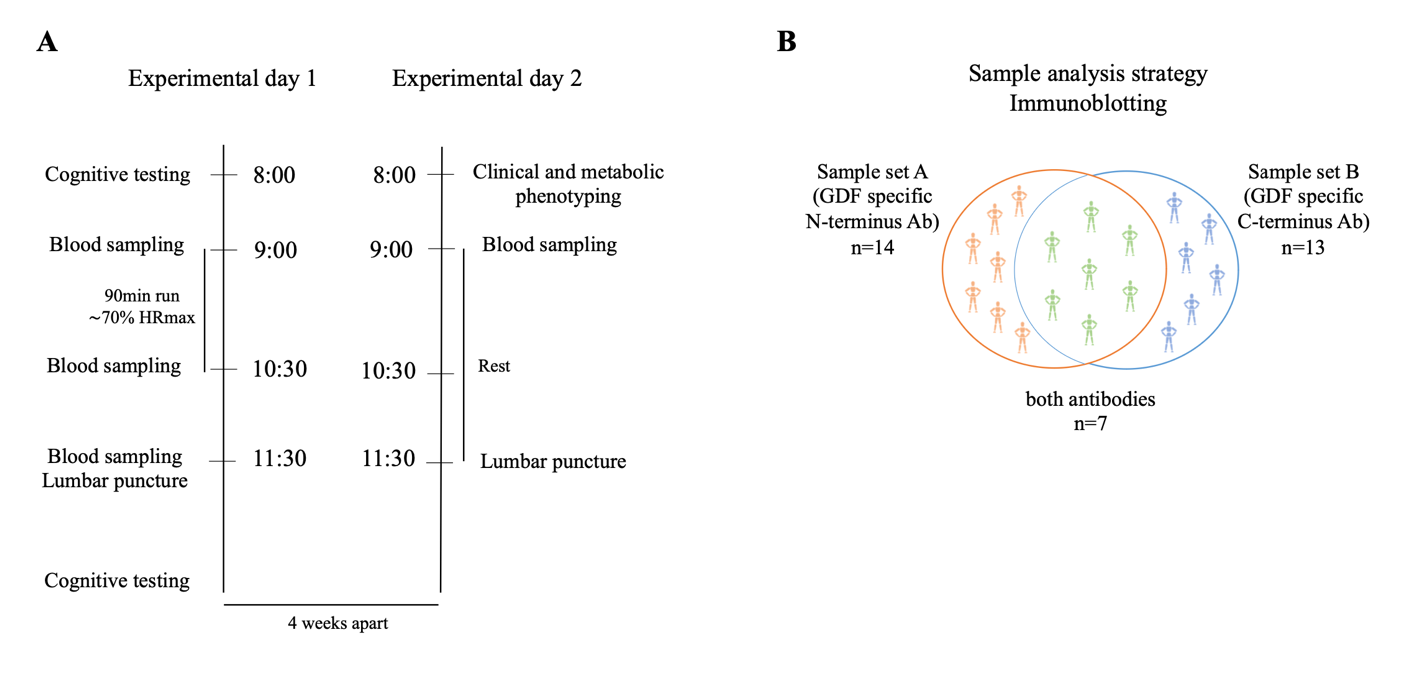


***Supplementary Figure 1.*** Study design (A); Sample analysis strategy (B). Due to the limited availability of cerebrospinal fluid (CSF), it was possible to test both GDF11-specific antibodies in the subset of paired CSF samples from 7 individuals.

**Supplementary Figure 2**


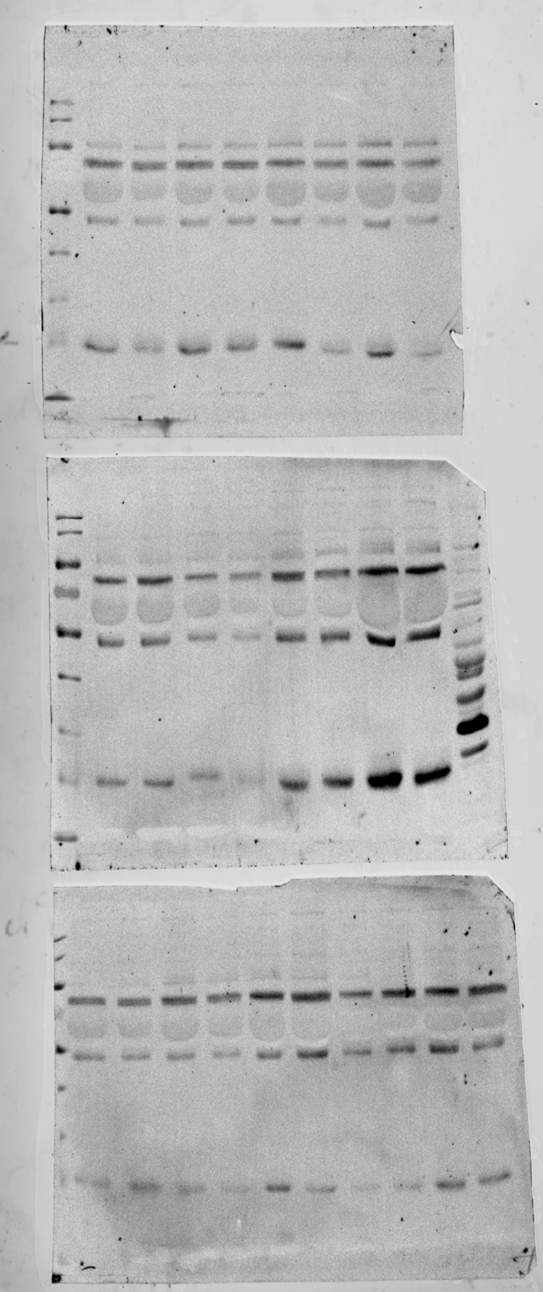


180

130

100

70

55

40

35

25

15


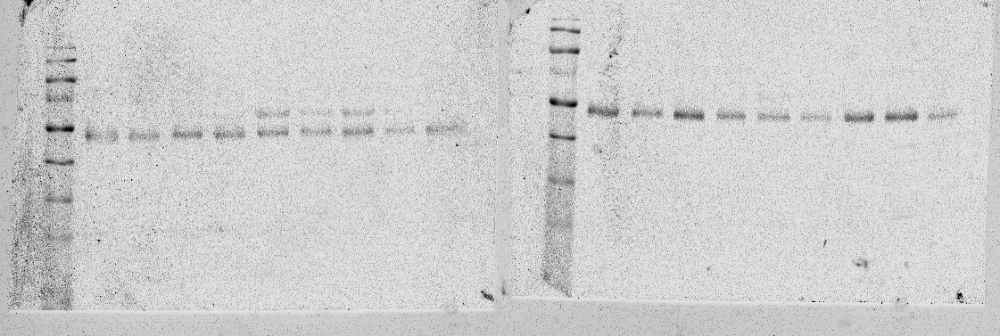

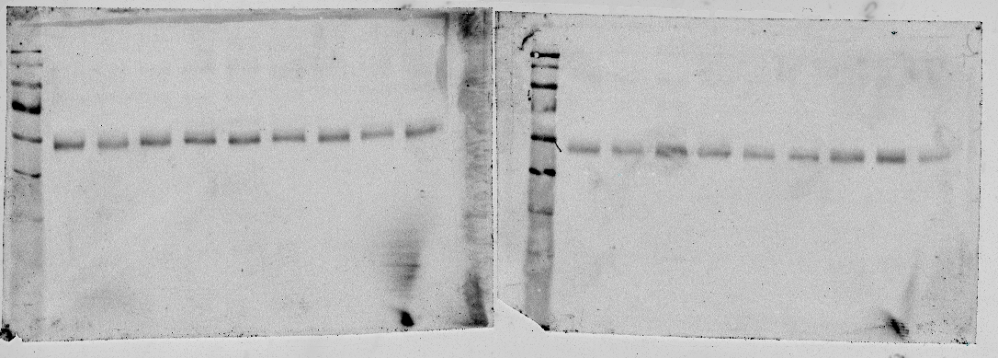


**A**

**B**

**C**

180

130

100

70

55

40

35

25

15

180

130

100

70

55

40

35

25

15

B R B R B R B R

B R B R B R B R

B R B R B R B R

Patient 11 Patient 14 Patient 16 Patient 18

Patient 18 Patient 19 Patient 21 Patient 22

Patient 11 Patient 14 Patient 15 Patient 16

B R B R B R B R

B R B R B R B R

B R B R B R B R

***Supplementary Figure 2.*** GDF11 in CSF assessed by immunoblotting, using three different primary antibodies: (A) GDF11-specific antibody against aa299-407 C-terminus; (B) GDF11 -specific antibody against aa31-66 N-terminus; (C) GDF11/GDF8 antibody against aa350-407 C-terminus.

**Supplementary Figure 3**

**
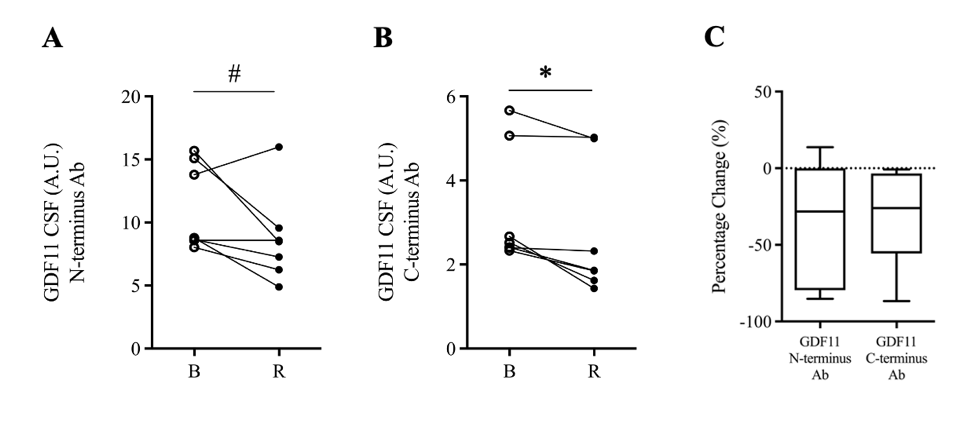
**

55

40


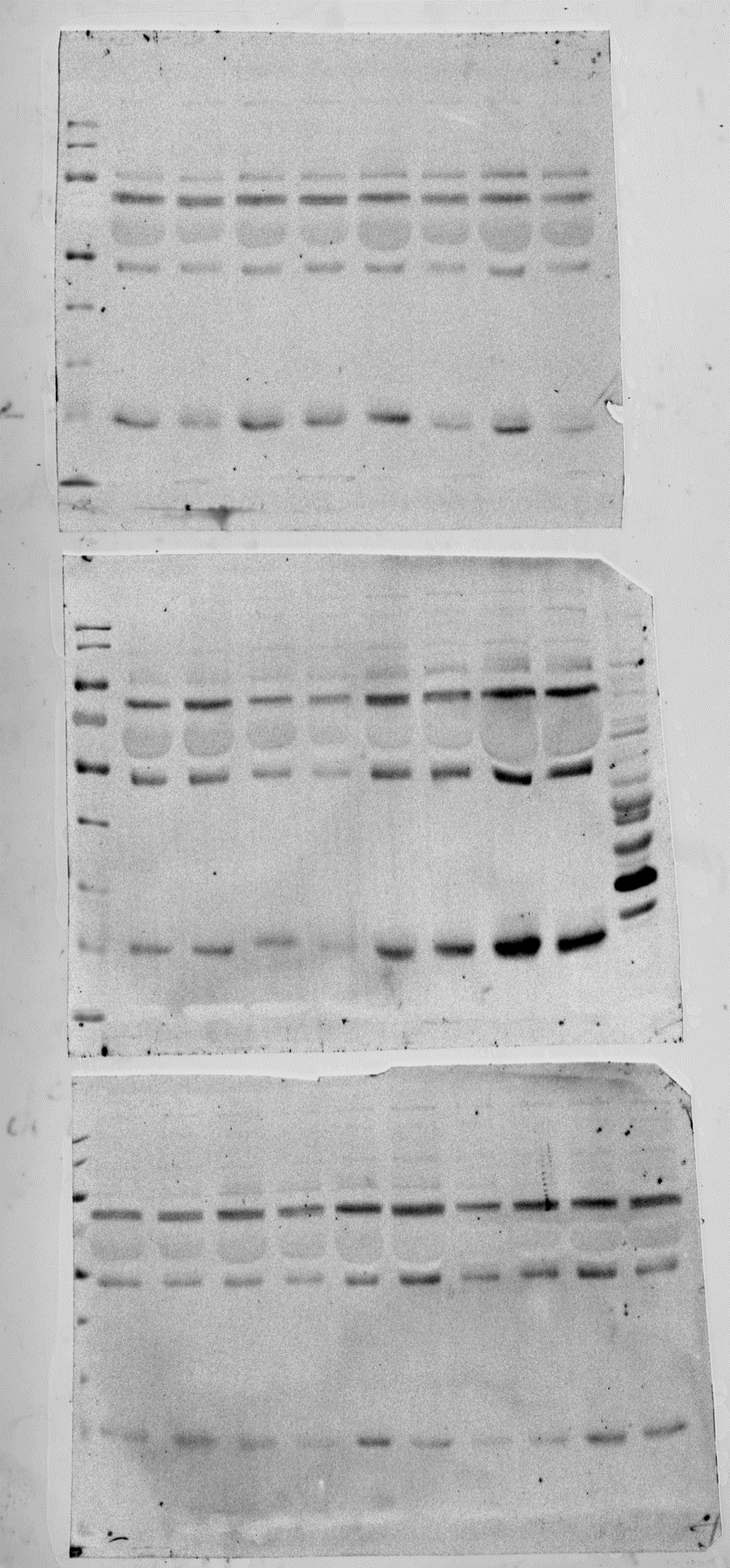

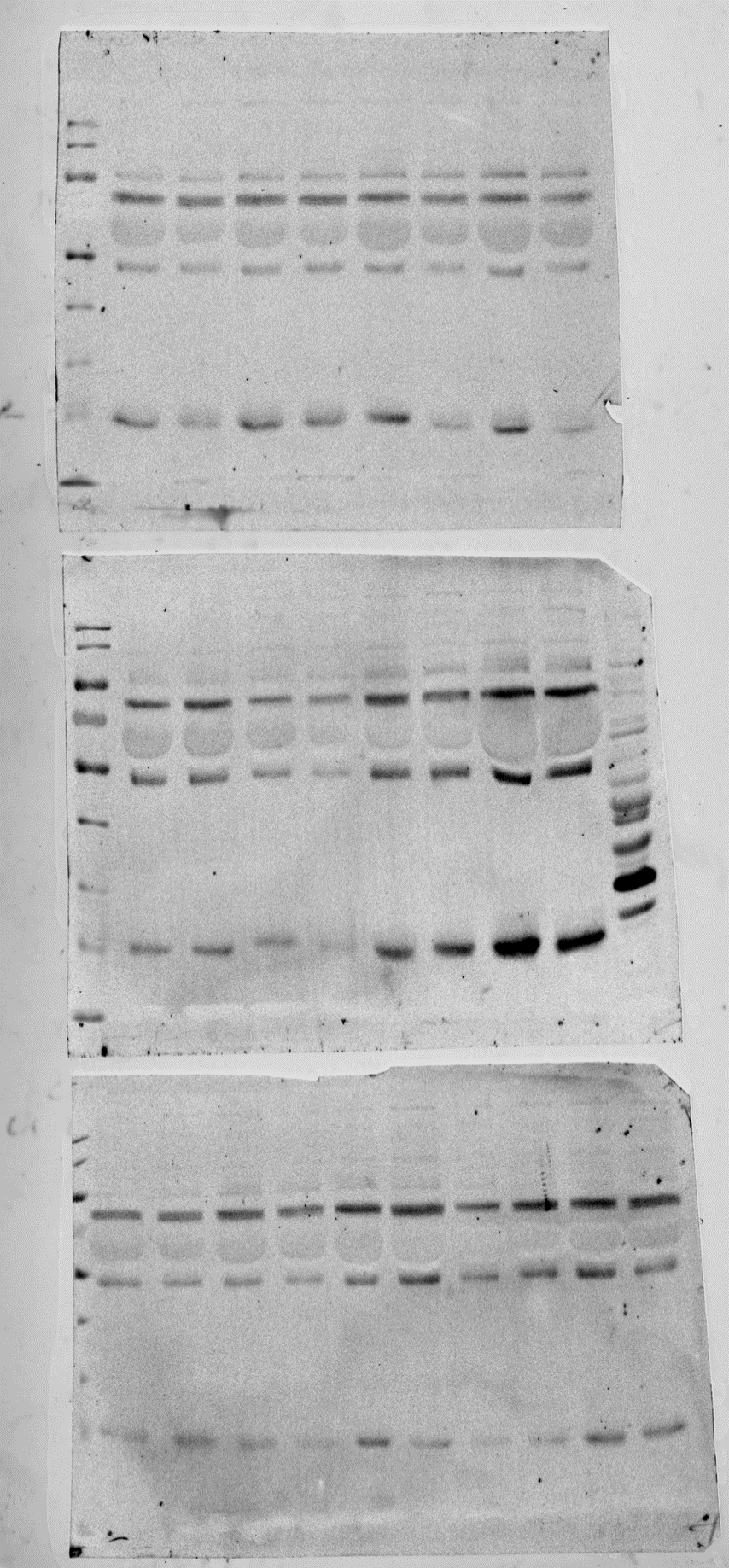


**D**

**E**

B R B R B R B R

B R B R B R

55

40


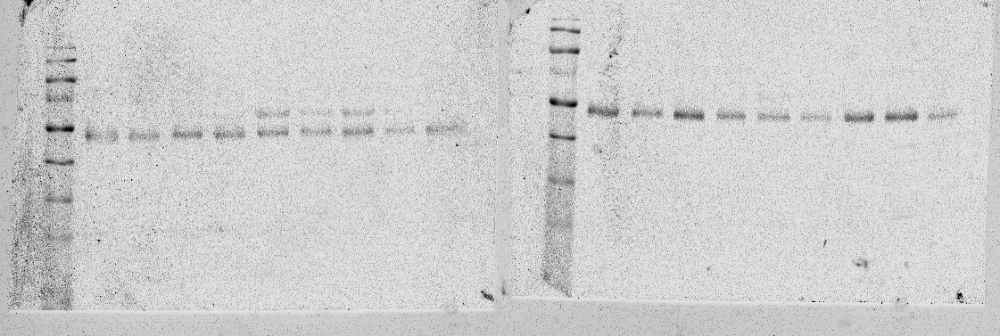

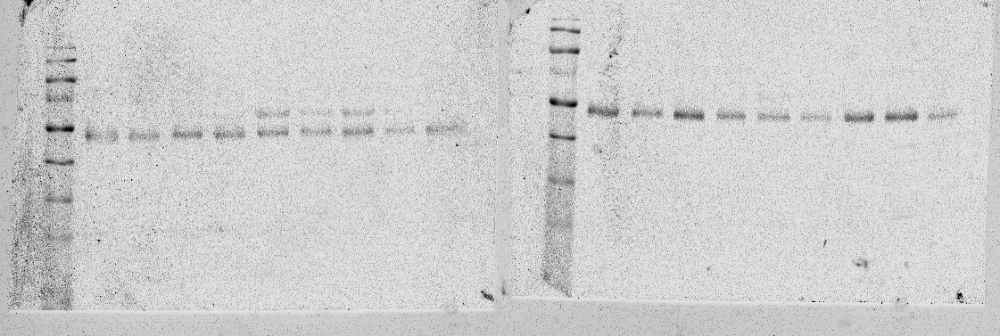


55

40

B R B R B R B R

B R B R B R B R

55

40

Patient 11 Patient 14 Patient 15 Patient 16

Patient 18 Patient 19 Patient 21 Patient 22

Patient 11 Patient 14 Patient 16 Patient 18

Patient 19 Patient 21 Patient 22

***Supplementary Figure 3.*** The comparison of GDF11 levels determined with two different antibodies in CSF of the same 7 individuals, before and after 90-min run. (A, E) GDF11-specific antibody against aa31-66 N-terminus; (B, D) GDF11-specific antibody against aa299-407 C-terminus; (C) Percentage change between antibodies. CSF, Cerebrospinal Fluid; GDF11, Growth Differentiation Factor 11. Statistical differences were analysed using paired Student’s t-test. *p<0.05, **p<0.01, # p<0.1.

**Supplementary Figure 4**


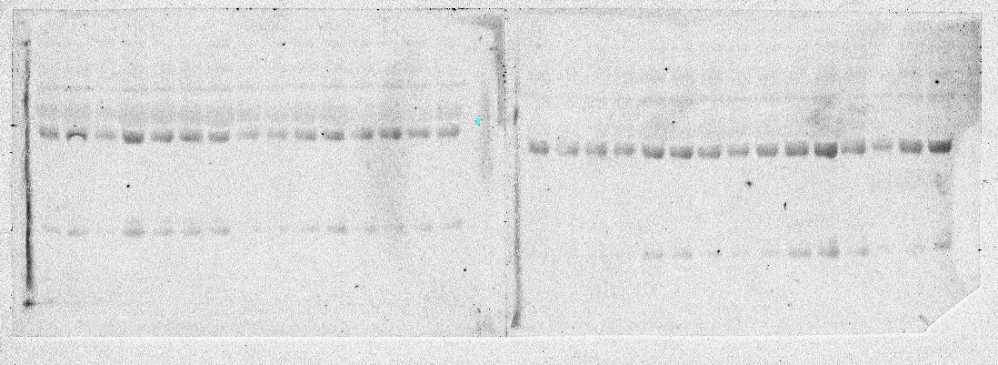

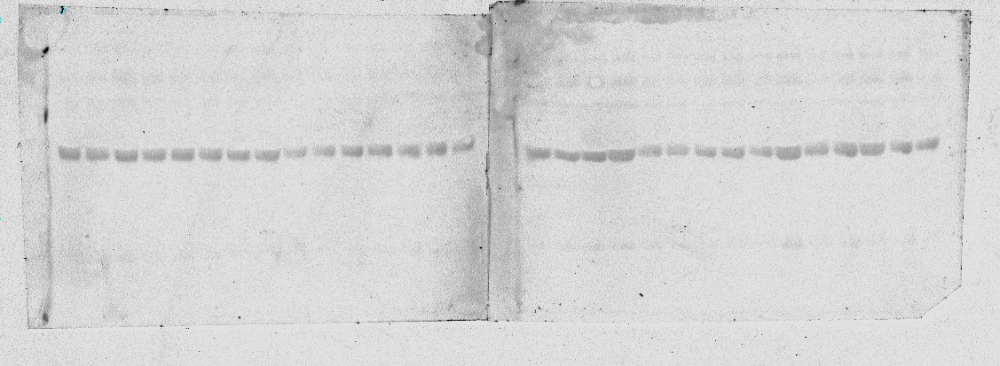


**A**

**B**

180

130

100

70

55

40

35

25

15

180

130

100

70

55

40

35

25

15

B 0 60 B 0 60 B 0 60 B 0 60 B 0 60

B 0 60 B 0 60 B 0 60 B 0 60 B 0 60

***Supplementary Figure 4.*** GDF11 in serum assessed by immunoblotting, using two primary antibodies: (A) GDF11-specific N-terminus antibody; (B) GDF11/8 antibody C-terminus ab. Due to the lack of space, molecular weight ladders are not clearly visible in Supplementary Figure 4 and therefore, the molecular weights of GDF11 are in this case approximate, based on our validation studies of antibodies. Immunoblotting of all samples was performed under identical conditions. GDF, Growth Differentiation Factor.
